# Do body mass index trajectories affect the risk of type 2 diabetes? A case–control study

**DOI:** 10.1186/s12889-015-2073-y

**Published:** 2015-07-28

**Authors:** Yoshihiko Mano, Hiroshi Yokomichi, Kohta Suzuki, Atsunori Takahashi, Yoshioki Yoda, Masahiro Tsuji, Miri Sato, Ryoji Shinohara, Sonoko Mizorogi, Mie Mochizuki, Zentaro Yamagata

**Affiliations:** Department of Sports and Exercise Nutrition, School of Physical Education, Sendai University, 2-2-18 Funaokaminami, Shibata, Miyagi Japan; Department of Health Sciences, Division of Medicine, Graduate School Department of Interdisciplinary Research, University of Yamanashi, 1110 Shimokato, Chuo City, Yamanashi Japan; Yamanashi Koseiren Health Care Center, 1-1-26 Iida, Kofu City, Yamanashi Japan; Center for Birth Cohort Studies, Graduate School Department of Interdisciplinary Research, University of Yamanashi, 1110 Shimokato, Chuo City, Yamanashi Japan; Department of Pediatrics, Division of Medicine, Graduate School Department of Interdisciplinary Research, University of Yamanashi, 1110 Shimokato, Chuo City, Yamanashi Japan; Nuffield Department of Primary Care Health Sciences, University of Oxford, Radcliffe Observatory Quarter, Woodstock Road, Oxford, UK

**Keywords:** Diabetes mellitus, Type 2, Body mass index, Aging, Blood glucose, Hyperglycemina, Hemoglobin A, Glycated, Metabolic syndrome X, General linear models, Case–control studies, Retrospective studies

## Abstract

**Background:**

Although obesity is a well-studied risk factor for diabetes, there remains an interest in whether “increasing body mass index (BMI),” “high BMI *per se*,” or both are the actual risk factors for diabetes. The present study aimed to retrospectively compare BMI trajectories of individuals with and without diabetes in a case–control design and to assess whether increasing BMI alone would be a risk factor.

**Methods:**

Using comprehensive health check-up data measured over ten years, we conducted a case–control study and graphically drew the trajectories of BMIs among diabetic patients and healthy subjects, based on coefficients in fitted linear mixed-effects models. Patient group was matched with healthy control group at the onset of diabetes with an optimal matching method in a 1:10 ratio. Simple fixed-effects models assessed the differences in increasing BMIs over 10 years between patient and control groups.

**Results:**

At the time of matching, the mean ages in male patients and controls were 59.3 years [standard deviation (SD) = 9.2] and 57.7 years (SD = 11.2), whereas the mean BMIs were 25.0 kg/m^2^ (SD = 3.1) and 25.2 kg/m^2^ (SD = 2.9), respectively. In female patients and controls, the mean ages were 61.4 years (SD = 7.9) and 60.1 years (SD = 9.6), whereas the mean BMIs were 24.8 kg/m^2^ (SD = 3.5) and 24.9 kg/m^2^ (SD = 3.4), respectively. The simple fixed-effects models detected no statistical significance for the differences of increasing BMIs between patient and control groups in males (*P* = 0.19) and females (*P* = 0.67). Sudden increases in BMI were observed in both male and female patients when compared with BMIs 1 year prior to diabetes onset.

**Conclusions:**

The present study suggested that the pace of increasing BMIs is similar between Japanese diabetic patients and healthy individuals. The increasing BMI was not detected to independently affect the onset of type 2 diabetes.

## Background

The worldwide population of individuals with diabetes is more than 382 million people [1], and potentially, 20.5 million of these are in Japan [2]. Because the subsequent ischemic heart disease, cerebral infarction, retinopathy, nephropathy, and neuropathy are life threatening to numerous people, the prevention of diabetes is of great importance. Among many environmental factors associated with diabetes, obesity is a particularly well-known risk factor for type 2 diabetes [3–5]. Notably, studies indicate that East Asian individuals with body mass indexes (BMIs) within normal range are much more likely to develop diabetes compared with Caucasians and African Americans with those BMIs [6].

Several studies have indicated that individuals with high BMIs are at a high risk of developing diabetes,[7–13]; however, due to their cross-sectional or 2–3 time-point cohort designs, these reports have not distinguished the risk of “increasing BMI” on diabetes onsets from that of “high BMI *per se.*” The present study from Japanese large-scale clinical data was driven by the interest in comparing BMI trajectories in diabetic patients and healthy individuals and by the question of whether increasing BMI alone would cause diabetes.

## Methods

### Ethics statement

This study was approved by the Ethical Review Board of the Faculty of Medicine, University of Yamanashi (approval number: H22 No.620). Approval was based on the ethical guidelines and regulations of the Declaration of Helsinki. The Japanese guidelines permit the use of data from medical check-ups without consent if the data are anonymous. Hence, informed consent was not required in this study because the data were obtained anonymously from observational check-up data. In addition, in Japan, subjects are not required to approve or disapprove the use of anonymous information from medical check-up data for research. All data were analyzed anonymously, and thorough care was taken to prevent assessed individuals from suffering any disadvantages.

### Study subjects

Data were from 64,762 individuals who underwent comprehensive health check-ups during the period from April 1, 1999 to March 31, 2009 at the Yamanashi Koseiren Health Care Center. The criteria for inclusion were undergoing at least two health check-ups over the 5-year period from April 1999 to March 2004. During the period, glycated hemoglobin A1c (HbA1c) and fasting plasma glucose (FPG) levels of the study subjects should not meet the Japanese diagnostic criteria for prediabetes of HbA1c ≥ 6.5 % or FPG ≥ 126 mg/dL (7.0 mmol/L) [14]. In addition to the criteria, the subjects were eligible if they underwent at least one check-up during the period from April 2004 to March 2009.

### Patient and control definitions

Patients were defined as subjects who were newly diagnosed with diabetes at other hospitals outside or who exhibited HbA1c ≥ 6.5 % or FPG ≥ 126 mg/dL during the period from April 2004 to March 2009. Control healthy subjects, whose HbA1c and FPG levels did not exceed the diagnostic criteria for diabetes within the 10-year survey period, were matched to diabetic patients for BMI and age at a 1:10 ratio at the time of diabetes onset of patients (time point 0). These operations were all performed separately for males and females. The diagnostic criteria were also based on the guidelines for epidemiologic research on diabetes by “Committee Report Regarding Diabetes Classifications and Diagnostic Criteria (internationally standardized version)” [15]. Matching was performed using an optimal matching [16].

### Definitions of time points

The year of diabetes onset in the patient group was set as time point 0. Each year was then measured with reference to time point 0. Thus, 1 year before onset was time point −1, and 2 years before onset was time point −2. This was continued to time point −9. Accordingly, time points are expressed in years.

### Clinical parameters

Clinical parameters included in our analysis comprised sex, date of birth, height, weight, HbA1c, and FPG levels. Age was calculated from date of birth. BMI was calculated as weight in kilograms divided by square of height in meters.

### Statistical analysis

We calculated mean and standard deviation (SD) values for age, weight, height, BMI, HbA1c, and FPG levels for each group at the matched time point. Student’s *t* test compared baseline characteristics between patient and control groups. As the major focus of this study, BMI trajectory was subjected to regression analysis, using a linear mixed-effects model that included time points, group categories, and interaction terms as explanatory categorical variables. The group categories included diabetic patients and healthy subjects, and the interaction terms were defined between time points and group categories. The past histories of the patient and control groups were reviewed, and mean BMIs were estimated using the linear mixed-effects model, from which a trajectory of each group was drawn, stratified for sex. With the aim of taking individual variability into consideration, a random effect for individuals was set for the intercept of the linear mixed-effects models. To assess whether increasing BMI affects the risk for diabetes, *P* value for the difference in increasing BMIs between patient and control groups was evaluated in another simple fixed-effects model with interaction terms that categorizes patient or control group and time points 0 or −9. All statistical analyses were performed using SAS version 9.3 (Cary, NC, USA). All reporte*P* values are two-sided; *P* values of <0.05 were considered to be statistically significant.

## Results

**Fig. 1:**
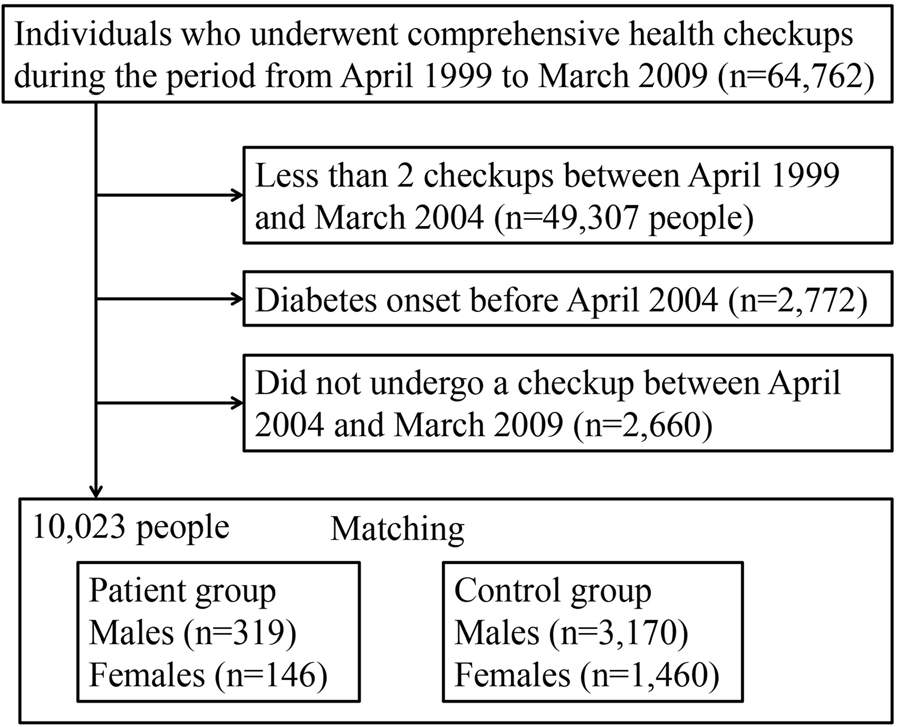
Flowchart of study individuals

Figure 1 shows how eligible and matched control subjects were selected. Table 1 represents the baseline characteristics in patient and control groups. The mean ages of males at the matching time point 0 were 59.3 (SD = 9.2) years in the patient group and 57.7 (SD = 11.2) years in the control group (*P* = 0.003), whereas the mean BMIs were25.0 (SD = 3.1) kg/m^2^ in the patient group and 25.2 (SD = 2.9) kg/m^2^ in the control group (*P* = 0.22), respectively. In females, the mean ages were 61.4 (SD = 7.9) years in the patient group and 60.1 (SD = 9.6) years in the control group (*P* = 0.06), whereas the mean BMIs were 24.8 (SD = 3.5) kg/m^2^ in the patient group and 24.9 (SD = 3.4) kg/m^2^ in the control group (*P* = 0.67), respectively.

**Table 1:** Characteristics of diabetic patients and healthy subjects at the matching time of diabetes onset

	Males			Females		
Variables, mean (SD)	Case (*n* = 319)	Control (*n* = 3170)	*P* for difference	Case (*n* = 146)	Control (*n* = 1460)	*P* for difference
Age, years	59.3 (9.2)	57.7 (11.2)	0.003	61.4 (7.9)	60.1 (9.6)	0.06
Weight, kg	69.9 (11.1)	70.8 (10.0)	0.13	57.6 (9.8)	59.3 (9.4)	0.047
Height, m	1.67 (0.07)	1.67 (0.06)	0.15	1.52 (0.06)	1.54 (0.06)	0.001
BMI, kg/m2	25.0 (3.1)	25.2 (2.9)	0.22	24.8 (3.5)	24.9 (3.4)	0.67
HbA1c, %	6.3 (0.6)	5.4 (0.3)	<0.0001	6.4 (0.8)	5.5 (0.3)	0.0001
FPG, mg/dL	130 (13)	101 (8)	<0.0001	129 (24)	98 (9)	0.0001

SD, standard deviation; BMI, body mass index; HbA1c, glycated hemoglobin A1c; FPG, fasting plasma glucose

**Fig. 2:**
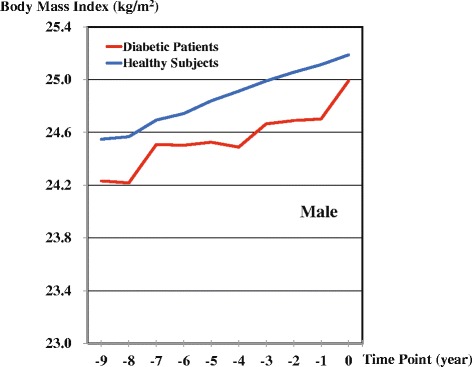
Retrospective BMI trajectories of patient and control groups by a linear mixed-effects model: Males

**Fig. 3:**
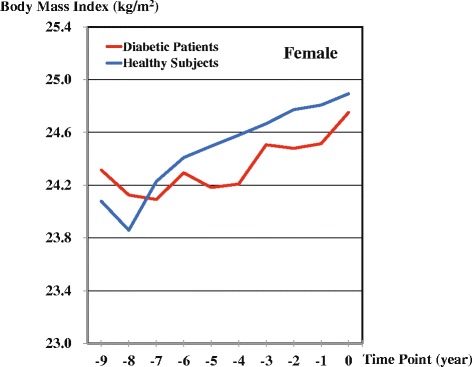
Retrospective BMI trajectories of patient and control groups by a linear mixed-effects model: Females

Table 2 and Figs. 2 and 3 show the trajectories in patient and control groups. In males, the mean BMI was consistently higher in the control group than in the patient group. In females, BMI also tended to be higher in the control group than in the patient group. In contrast to males, a lesser increase in BMI was observed over the nine years in the female patient group than in the female control group. Sudden increases in BMI were observed in both male and female patients from one year before diabetes onset. Statistically significant differences in BMI as interaction terms were observed between the patient and control groups at −1 (*P* = 0.01), −2 (*P* = 0.01), −3 (*P* = 0.045), and −4 (*P* = 0.0002) time points among males and at −4 (*P* = 0.03), and −8 (*P* = 0.04) time points among females. The simple fixed-effects models, which examined the major question of this study, represented *P* of 0.19 in males and 0.67 in females between the increasing BMIs in patient and control groups.

**Table 2:** Estimated mean BMIs before onsets of diabetes based on coefficients in mixed-effects models in patients and matched healthy subjects

Time Points	−9	−8	−7	−6	−5	−4	−3	−2	−1	0
Male										
Diabetic patients	24.23	24.22	24.51	24.50	24.53	24.49	24.66	24.69	24.70	24.99
Healthy subjects	24.55	24.57	24.69	24.74	24.84	24.91	24.99	25.06	25.11	25.19
Female										
Diabetic patients	24.31	24.12	24.09	24.29	24.18	24.21	24.51	24.48	24.51	24.75
Healthy subjects	24.08	23.86	24.23	24.41	24.50	24.58	24.67	24.77	24.81	24.89

## Discussion

The results of this study suggest that the retrospective trajectories of BMIs in patients with newly-acquired diabetes are similar to those in healthy individuals. To the best of our knowledge, this is the first study comparing BMI trajectories between Japanese individuals with and without diabetes using repeated data.

Vistisen et al. described large changes in BMI in patients with diabetes in the UK without using matching case to control [17], although typical BMI for the onset of type 2 diabetes greatly differed between individuals of East Asian descent and individuals of European descent [18]. Therefore, the present data may be specific to a type of Japanese diabetic patients and hence may result in limited generalizability. No previous reports described sudden increases in BMI prior to diabetes onset. One Japanese study, although performed over two time points, reported that the risk for diabetes onset increased when either BMI or body weight increased [19]. In a previous study, Kodama et al. demonstrated that individuals of East Asian descent with non-overweight or slightly overweight BMIs also have a high risk of developing type 2 diabetes [6]. These studies are consistent with our results presenting increasing but almost normal-ranged BMIs among the diabetic patients.

The most likely mechanism for the diabetic onsets in patient group is insulin resistance and impaired insulin secretion in Japanese individuals with moderately high and increasing BMI. According to a previous systematic review, individuals of East Asian descent, which includes Japanese people, have higher insulin sensitivity than people from Western countries, whereas the insulin secretory ability is low only in East Asian individuals with diabetes. It has been reported that when the body weight of an individual with impaired insulin secretion increases, the risk of developing type 2 diabetes also increases [6]. It appears that this mechanism is involved in diabetes onset in patients in the present study. In another study that identified a polymorphism involved in the onset and progression of type 2 diabetes, the KCNJ15 polymorphism was observed in many non-overweight diabetic patients of Japan, whose ability to secrete insulin tended to decrease over time [20]. Although the patients and healthy subjects in this study had not undergone genetic testing, increased body weight in such individuals with decreased insulin secretory ability may have increased insulin resistance [21, 22] and it may have increased their susceptibility to developing diabetes. Another pathway of diabetic onset may be attributed to the observed fluctuation in BMI prior to the onset of diabetes in this study. Because literature suggests that weight fluctuation increases the risk of type 2 diabetes in American women [23], the observed fluctuation among the patient group may have affected their insulin resistance [24]. Alternatively, the observed fluctuation may reflect the small sample sizes of the male and female patient groups.

Literature is available on the sensitivity and specificity for an HbA1c threshold of 6.5 %. Ito reports a sensitivity of 53.4 % and a specificity of 94.6 % for an HbA1c threshold of 6.5 % in a Japanese population [25]. In a Chinese population, Bao et al. report a sensitivity of 53.7 % and a specificity of 97.4 % for an HbA1c threshold of 6.5 % [26]. Because Choi et al. report a sensitivity of 52 % and a specificity of 97 % for an HbA1c threshold of 6.2 % in a Korean population, the sensitivity for an HbA1c threshold of 6.5 % should be lower than 52 % [27]. Furthermore, the latest data from the American National Health and Nutrition Examination Survey describes a sensitivity of approximately 44 % and a specificity of approximately 98 % at an HbA1c threshold of 6.5 % [28]. On the other hand, data from a Japanese population has reported a sensitivity of 86.5 % and a specificity of 87.3 % at the FPG threshold of 115 mg/dL (6.4 mmol/L) [29]. Thus, the present epidemiologic-research-based diagnosis for diabetes by the combination of HbA1c ≥ 6.5 % or FPG ≥ 126 mg/dL [15] would have achieved an acceptable sensitivity and specificity.

The strengths of this study include the fact that BMI trajectories of both patients and controls were matched for the timing of diabetes onset, the BMI, and the age. Thus, this study is considered to have excluded the effect of having high BMI *per se* on the risk of developing diabetes. Additionally, we used chronological data in which BMIs were measured at three or more time points. Although subjects were not identical at each time point, it is noteworthy that the data depicted the trajectories over 10 time points.

A limitation of this study is that, in addition to the interviews with the patients regarding their newly-acquired diabetes, diabetes was partly diagnosed based on HbA1c and FPG levels rather than on a confirmed diagnosis from secondary tests or glucose tolerance tests. Although we used criteria equivalent to parts of the diagnostic criteria used by physicians in Japan [14], and guidelines for epidemiologic research on diabetes allow for the use of the criteria applied in the present study [15], it is possible that some subjects were incorrectly classified as patient group subjects. Results in Tables 1 and 2 show older ages and larger weight gains from the previous year in the patient group than the control group. Because people who are either older or are gaining weight are more likely to undergo health check-ups and be diagnosed with diabetes [30], the misclassification may have been differential and hence may have produced an associated bias toward broadening the difference in BMI trajectories between patient and control groups. The second limitation of our study was that all subjects underwent a paid comprehensive health check-up. Therefore, these results may not be directly applicable to the entire Japanese population. Another limitation was the fact that because this study used retrospective data from the time of disease onset (time point 0), the population was not identical at each observational time point. However, this had probably only little effect, as no marked generational changes were seen over the period from 1999 to 2009 in attitudes toward health, diet, and exercise habits of Japanese people. Furthermore, although adjustment by multivariate regression for confounding factors could be employed in this study, we have exploited simple illustrations to compare trajectories, matching patients and healthy subjects with confounding factors.

This study suggests that the body weight of some patients with type 2 diabetes increases immediately before disease onset. Therefore, healthy but pre-diabetic individuals whose body weight has been increasing for the past few years should receive proactive guidance regarding diet and exercise from their physicians, and they should be encouraged to control their body weight. The gradually increasing mean BMI of individuals with and without diabetes in our study reached approximately 25 at the final measurement. This suggests that even if BMI is within normal range (18.5–25), physicians should be recommended to strongly advise East Asian adults not to gain any weight.

## Conclusions

The present study from large-scale clinical data of Japanese residents suggests that the pace of increasing BMI in diabetic patients is similar to that in healthy individuals. The results also indicate that the BMIs of the Japanese patients do not markedly increase prior to the onset of type 2 diabetes.

## Abbreviations

BMI: Body mass index
HbA1c: Glycated hemoglobin A1c
FPG: Fasting plasma glucose
SD: Standard deviation
